# Chemistry of Fluorinated Pyrimidines in the Era of Personalized Medicine

**DOI:** 10.3390/molecules25153438

**Published:** 2020-07-29

**Authors:** William H. Gmeiner

**Affiliations:** Department of Cancer Biology, Wake Forest School of Medicine, Winston-Salem, NC 27157, USA; bgmeiner@wakehealth.edu; Tel.: +1-336-716-6216

**Keywords:** fluoropyrimidine, thymidylate synthase, DNA topoisomerase 1, DNA repair, pseudouridine, ribothymidine

## Abstract

We review developments in fluorine chemistry contributing to the more precise use of fluorinated pyrimidines (FPs) to treat cancer. 5-Fluorouracil (5-FU) is the most widely used FP and is used to treat > 2 million cancer patients each year. We review methods for 5-FU synthesis, including the incorporation of radioactive and stable isotopes to study 5-FU metabolism and biodistribution. We also review methods for preparing RNA and DNA substituted with FPs for biophysical and mechanistic studies. New insights into how FPs perturb nucleic acid structure and dynamics has resulted from both computational and experimental studies, and we summarize recent results. Beyond the well-established role for inhibiting thymidylate synthase (TS) by the 5-FU metabolite 5-fluoro-2′-deoxyuridine-5′-*O*-monophosphate (FdUMP), recent studies have implicated new roles for RNA modifying enzymes that are inhibited by 5-FU substitution including tRNA methyltransferase 2 homolog A (TRMT2A) and pseudouridylate synthase in 5-FU cytotoxicity. Furthermore, enzymes not previously implicated in FP activity, including DNA topoisomerase 1 (Top1), were established as mediating FP anti-tumor activity. We review recent literature summarizing the mechanisms by which 5-FU inhibits RNA- and DNA-modifying enzymes and describe the use of polymeric FPs that may enable the more precise use of FPs for cancer treatment in the era of personalized medicine.

## 1. Introduction

Medicinal applications of fluorinated drugs continue to expand rapidly, in part because of new developments in fluorine chemistry that extend the range of compounds that can readily be prepared with fluorine substitution, and because of the increased understanding of how biological and biochemical processes are uniquely perturbed by fluorine substitution. The effects of fluorine on the biological activities of drug-like molecules result, in part, from fluorine’s high electronegativity but low propensity to engage in hydrogen bond formation. What is also important is the strength of the C–F bond and the relatively small size of fluorine relative to other potential substituents. For example, the strength of the C–F bond is critical to FdUMP inhibiting thymidylate synthase (TS), as dUMP analogs that include halogens with weaker C–X bonds undergo dehalogenation by TS, while FdUMP remains stably bound inhibiting further enzymatic activity.

A key concept in the use of fluorinated analogs of native metabolites is lethal synthesis [1]. Lethal synthesis involves biological transformation of a relatively non-toxic metabolite into a more toxic form. For example, fluoroacetate, which is relatively non-toxic, is converted to fluorocitrate, which inhibits two enzymes of the Krebs cycle (aconitase and succinic dehydrogenase), and is highly toxic to mammalian cells. Capitalizing on the increased uptake of uracil by some malignant cells, Heidelberger developed fluorinated pyrimidines (FPs) [2], and showed that 5-fluorouracil (5-FU) is metabolized to compounds that are highly cytotoxic to a variety of cells including cancer cells [3]. While most enzymatic reactions that use uracil or uridine derivatives as substrates proceed with similar kinetics with the fluoro analog as for the native substrate [4,5], notable exceptions were identified. In particular, thymidylate synthase (TS) was inhibited by the 5-FU metabolite, FdUMP [6]. TS inhibition causes cancer cells reliant on the de novo thymidylate pathway to undergo thymineless death [7].

**Figure 1:**
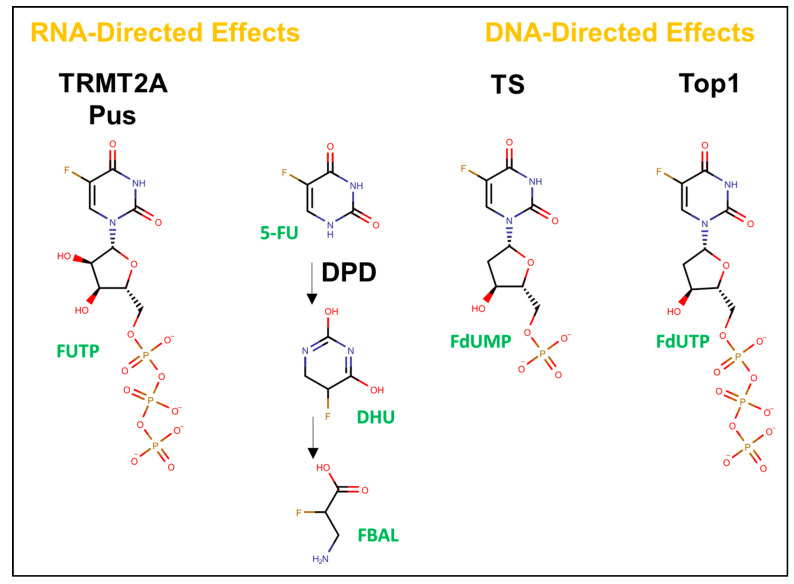
Overview of 5-FU metabolites and enzymatic targets of potential interest for personalized medicine applications. 5-FU is metabolized to dihydrouracil (DHU) by dihydropyrimidine dehydrogenase (DPD). Inter-patient variability in DPD activity makes appropriate dosing of 5-FU challenging. DNA-directed effects of 5-FU are due to FdUMP, which inhibits thymidylate synthase (TS) and FdUTP that is misincorporated into DNA leading to the poisoning of DNA Topoisomerase 1 (Top1). FUTP becomes misincorporated into RNA and inhibits Urd-modifying enzymes including TRMT2A and pseudouridine (Ψ) synthase (Pus). A more complete depiction of the 5-FU metabolism appears in [15].

Advances in fluorine chemistry enabled the synthesis of 5-FU on an industrial scale [8], which contributed to the widespread adoption of FP drugs for the treatment of colorectal cancer (CRC). 5-FU is currently used to treat >2 million cancer patients worldwide each year [9]. Furthermore, TS inhibition is one of the best-validated and most successful strategies ever used for cancer treatment [10]. Concomitant with the increased availability of 5-FU and FP drugs were increased investigations into mechanisms beyond TS inhibition [11] that are important for the anti-cancer activities of FP drugs, which include the poisoning of DNA topoisomerase 1 (Top1; [12,13]). Furthermore, while the importance of RNA-mediated processes for 5-FU’s systemic toxicities was established decades ago, the discovery of specific RNA-mediated processes perturbed by RNA remains an active area of research. In this regard, potential new roles for RNA modifying enzymes perturbed by 5-FU [14], including tRNA methyltransferase 2 homolog A (TRMT2A) and pseudouridine synthase (Pus), are under investigation (Figure 1).

A major challenge today is how to customize drug use to the genetic profile of individualized patients, i.e., implement personalized medicine [16]. The enzymes that are of particular significance for 5-FU activity that display particularly large inter-patient variability include dihydropyrimidine dehydrogenase (DPD; encoded by the *DPYD* gene; [17]) and TS (encoded by *TYMS*; [18]). The chemistry of FPs has evolved to address this variability. Patients who are deficient in 5-FU catabolism due to polymorphisms in *DPYD* are exceedingly sensitive to 5-FU and at high risk for serious toxicities due to treatment [19]. This has resulted in the development of DPD modulators [20], but also synthesis of isotopically enriched 5-FU, to quantify catabolism in vivo. The importance of TS inhibition for FP activity has resulted in chemical methods for synthesis of FP polymers that display both improved anti-tumor activity and reduced systemic toxicities [21]. We review literature related to expanding the chemistry of FPs for improved use in the era of personalized medicine.

## 2. Synthesis and Isotopic Labeling of 5-FU

The initial synthesis of 5-FU that was reported by Heidelberger and co-workers in 1957 [22] was based on a modification to a ring closure approach developed for pioneering studies in pyrimidine chemical synthesis. Briefly, this approach involved reacting isothiourea salts with α-fluoro-β-ketoester enolates to generate the pyrimidine skeleton already substituted with fluorine. A disadvantage of the ring closure route to 5-FU synthesis was the high toxicity of the ethyl fluoroacetate reagent. This resulted in a search for alternative synthetic routes that were less likely to be hazardous to laboratory personnel. Barton and co-workers were the first to develop a procedure for the electrophilic fluorination of uracil and demonstrated specific monohalogenation at the 5 position using fluoroxytrifluoromethane (CF_3_OF) [23]. Electrophilic fluorination is a process by which fluorine is delivered to an electron-rich reactant, such as an alkene, aromatic ring, or carbanion, by a formal “positive-fluorine” reagent, such as quaternary ammonium R3N+F A- salts, to form a carbon-fluorine covalent bond. Modified approaches that used electrophilic substitution to prepare 5-FU were reported by others, and such approaches were used for commercial-scale 5-FU production [8], which contributed to the widespread clinical adoption of 5-FU in oncology. An important advance in using electrophilic substitution for 5-FU synthesis was made by using SelectFluor^TM^ (F-TEDA-BF_4_; 1-chloromethyl-4-fluoro-1,4-diazoniabicyclo [2.2.2]octane bis(tetrafluoroborate)–an *N*-fluoroammonium salt electrophilic fluorinating agent containing an N–F bond. SelectFluor^TM^ was developed to overcome limitations of other reagents used for electrophilic fluorine substitution that were more difficult to handle [24], and SelectFluor^TM^ was demonstrated to provide a practical and direct route to 5-FU synthesis [25].

**Figure 2:**
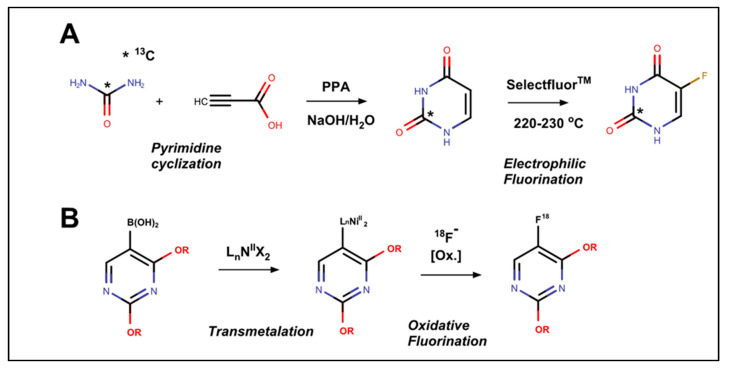
Strategies for the synthesis of labeled 5-FU for personalized medicine studies. (**A**) Cyclization approach starting from [^13^C] urea and propiolic acid. Treatment with polyphosphoric acid (PPA), and then sodium aqueous yields [2-^13^C] uracil. Electrophilic fluorination with Selectfluor yields ^13^C-labeled 5-FU for metabolism studies (adapted from [29]). (**B**) Transmetalation approach for introducing ^18^F Fluoride. The Nickel σ-aryl complex of protected uracil was generated from the corresponding boronic acid precursor and reacted with [^18^F] fluoride and oxidant to yield ^18^F-labeled 5-FU for positron emission tomography (PET) imaging studies (adapted from [26]).

More recently, studies related to 5-FU synthesis have focused on incorporating stable or radioactive isotopes for biological studies or to enable personalized therapy (Figure 2). For example, to enable positron emission tomography (PET) imaging of 5-FU, a transmetalation reaction was developed in which an arylboronic acid precursor of 5-FU was first converted to a Nickel(II) σ-aryl complex, which was then reacted with [^18^F]fluoride to yield [^18^F]5-FU [26]. The availability of an improved synthetic route to [^18^F]5-FU should enable future studies into the biodistribution of 5-FU and its metabolites in humans [27] and laboratory animals [28]. This chemical approach should be applicable to 5-FU analogs and could guide the use of new agents with improved biodistribution profiles and activities.

Isotopic enrichment of 5-FU, especially at C2, is important for following the degradation of 5-FU in vivo because 5-FU catabolism results in the loss of the C2 carbonyl as carbon dioxide (CO_2_). Thus, [2-^13^C]- and [2-^14^C]-5-FU degradation kinetics in vivo can be followed by the release of ^13^CO_2_ or ^14^CO_2_ from 5-FU treated subjects. Such studies are of particular importance for the use of 5-FU in the era of personalized medicine because a significant percentage of cancer patients are deficient in 5-FU degradation due to polymorphisms in *DPYD* [30], the gene encoding dihydropyrimidine dehydrogenase (DPD), which catalyzes the initial step in 5-FU degradation. As a result, patients with DPD deficiencies experience serious toxicities that are occasionally lethal. C2 labeling of Ura was accomplished starting from ^13^C urea by cyclization upon reacting labeled urea with propiolic acid. The resulting [2-^13^C] Ura was then converted to [2-^13^C]5-FU using Selectfluor^TM^ [29]. [2-^13^C]5-FU can be used to detect patients with deficiencies in 5-FU metabolism by detecting ^13^CO_2_ by mass spectrometry analysis of exhaled air (i.e., “breath test”). Such a chemically-oriented assay may be a more general approach to detecting the sensitive patient population since multiple genes may affect 5-FU metabolism [31], making genetic screening complex, expensive, and vulnerable to false negatives regarding 5-FU toxicity. In contrast, chemical approaches using DPD-inhibitory compounds, such as eniluracil, and 5-chloro-2,4-dihydroxypyridine (CDHP) to modulate 5-FU activity and toxicity have in general been disappointing [32]. DPD inhibitors have also been used in combination with orally bioavailable 5-FU analogs such as capecitabine [33] or tegafur [34] to provide a pharmacokinetic profile similar to continuous intra-venous (CIV) infusion of 5-FU. In general, this approach has not proven advantageous to CIV 5-FU, although it may be more convenient to implement.

While 5-FU is the most widely used FP for cancer treatment, other FPs have been synthesized and undergone biological evaluation. Synthesis of 6-fluorouracil was reported by Wempen and Fox [35], but this compound had minimal biological activity and new approaches to its synthesis have not been reported. The substantial difference in activity between the 5-Fluoro- and 6-Fluoro- Ura analogs results from the selective stabilizing effects of the 5-fluoro substituent to the Michael addition adduct in enzymatic reactions for which Ura is a substrate (summarized in Section 3: TS inhibition and DNA-directed effects of FPs). While not as potent a TS inhibitor as the 5-FU metabolite FdUMP, 5-(trifluoromethyl)-2′-deoxyridine 5′-monophosphate, which is a metabolite of trifluorothymine, forms a moderately stable covalent complex with TS [36]. Trifluorothymine was synthesized by Heidelberger and co-workers starting from trifluoroacetone, which was converted to the cyanohydrin, and used in a cyclization reaction with urea to generate the trifluoromethyl-substituted pyrimidine [37]. A more recent synthesis used catalytic trifluormethylation of uracil with CF_3_I in the presence of Fe(II) compounds [38]. This approach was applicable to both uridine and 2′-deoxyuridine making a separate glycosylation step unnecessary for preparation of trifluorthymidine (trifluridine) [38]. The direct preparation of trifluridine from 2′-deoxyuridine with trifluoromethyl sulfinate was also described [39] (CN104761602A; issued 2017). Trifluridine was recently approved for use in metastatic colorectal cancer by the U.S. Food and Drug Administration (FDA) as part of Lonsurf, which also includes Tiperacil, a thymidine phosphorylase (TP) inhibitor [40].

## 3. TS Inhibition and DNA-Directed Effects of FPs

**Figure 3:**
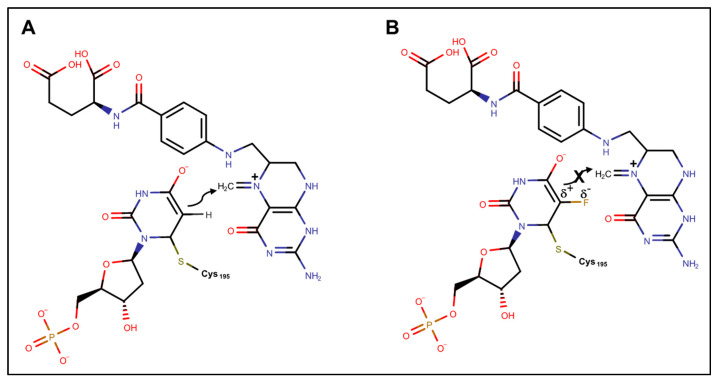
Depiction of the Michael adduct derived from Cys195 of human TS attack at C6 of (**A**) dUMP and (**B**) FdUMP upon interacting with the N5 iminium ion derived from N^5^,N^10^-methylene tetrahydrofolate. In (**A**) nucleophilic attack by C5 of the enolate results in C–C bond formation, while in (**B**) fluorine polarizes the C–F bond inhibiting C–C bond formation, and due to the strength of the C–F bond, dehalogenation does not occur and the adduct remains stably bound to TS.

Considerable evidence indicates TS inhibition and perturbation of DNA-mediated processes is primarily responsible for the anti-cancer activity of FPs [10,41]. The 5-FU metabolite FdUMP is a potent TS inhibitor [42]. The role of fluorine in TS inhibition by FdUMP is to stabilize the enolate formed upon Michael addition at C6 by a reactive Cys (C195 in human TS; Figure 3) [11]. For the normal substrate, dUMP, the formation of the Michael adduct stimulates binding of N^5^, N^10^-methylene tetrahydrofolate co-factor, which occurs with the formation of an N5 iminium ion. Bond formation proceeds by nucleophilic attack from C5 of dUMP enolate on the iminium ion; however, with fluorine stabilization, nucleophilic attack does not proceed, and a new C–C bond is not formed. While iodo- and bromo-analogs of FdUMP undergo dehalogenation of the enolate upon binding TS, the strength of the C–F bond results in the formation of a stable adduct. The ternary complex (TS/FdUMP/folate) may be detected by Western blot and is an indicator of TS inhibition [43]. Efforts to maximize the clinical efficacy of 5-FU focus on biochemical modulation by co-treatment with the folate analog Leucovorin [44], which promotes TS ternary complex formation despite the relatively low plasma folate levels of humans [45]. Continuous intra-venous infusion of 5-FU [46] is also used to maximize exposure to malignant cells while they are in S-phase when high TS levels occur. Therapeutic drug monitoring (TDM) is being implemented to optimize 5-FU plasma levels and account for high inter-patient variability in drug metabolism [47].

TS inhibition is central to the anti-tumor activity of FPs [10], and colorectal (CRC) tumors that respond to 5-FU were found to express low levels of TS and two other enzymes that affect 5-FU metabolism: DPD and thymidine phosphorylase (TP) [48]. Elevated intra-tumoral DPD limits levels of all anabolic 5-FU metabolites, while the TP-TK pathway [49] can either promote FdUMP formation from 5-FU or promote degradation of FdUMP that is formed via a multi-step process that requires orotic acid phosphoribosyl transferase (OPRTase), ribonucleotide reductase (RR), and other enzymes (e.g., OPRTase-RR pathway). The observed clinical dependence of 5-FU on low TP in mCRC [48] indicates that FdUMP production occurs predominantly via the OPRTase-RR pathway, and that in CRC cells, elevated TP primarily reduces FdUMP levels resulting in decreased TS inhibition.

Thus, FP analogs that are converted directly to FdUMP may be particularly effective anti-tumor agents by maximizing levels of the TS inhibitory metabolite FdUMP and circumventing pathways that increase the degradation of FP metabolites by DPD and/or TP. For example, a FdU phosphoramidate pro-drug of FdUMP was synthesized to enable direct intracellular formation of FdUMP [50]. Furthermore, the Gmeiner laboratory has developed FP polymers (e.g., F10 and CF10 [15]), and these can be directly converted to FdUMP [51] and retain strong activity towards cells that express elevated TS and are resistant to 5-FU [52]. The anti-tumor activity of FP polymers has been demonstrated in multiple pre-clinical models including acute leukemia [53,54], GBM [55], prostate cancer [56], and colorectal cancer [57]. The 2nd generation FP polymer CF10 includes additional chemical modifications to improve stability [15], and it has been selected for further study as a novel nanoscale material with strong potential for cancer treatment by the Nanotechnology Characterization Laboratory (https://ncl.cancer.gov/).

Topoisomerases are essential enzymes that regulate DNA topology during DNA replication and transcription [58]. While these enzymes are established targets for anti-cancer drugs, including camptothecin (CPT) and doxorubicin, there is increasing evidence that the DNA-directed activities of FPs occur by poisoning topoisomerase function, particularly of DNA Top1 [13]. Unlike the mechanism by which FPs inhibit TS, FdU inhibits Top1 by becoming incorporated into genomic DNA and interfering in the re-ligation step of Top1 catalysis [12]. Thus, Top1 efficiently cleaves DNA proximal to sites of FdU substitution, but the covalent Top1 cleavage complex (Top1cc) becomes trapped and does not undergo re-ligation [12,53]. Furthermore, the repair of FdU-dependent Top1cc displays a different dependence on Tdp1 and PARP1 [59], relative to CPT [60]. These findings indicate that FPs may cause replication stress by trapping Top1cc to block replication fork progression [61], and also by depleting cellular thymidylate [61]. Polymeric FPs such as F10 [62] and CF10 [15] that are more directly converted to FdUMP and FdUTP than 5-FU display improved anti-tumor activity and reduced systemic toxicities through dual targeting of TS and Top1 [12,53]. This approach shows promise for the treatment of malignancies with mutational profiles that limit the efficacy of conventional FPs, such as p53 mutations [63], and so may be important for a personalized medicine approach to treatment since many anti-cancer drugs, including 5-FU, are relatively less effective towards p53 mutant cancer cells [64].

Finally, it should be noted that a component of the DNA-directed effects of FPs may be due to 5-FU mutagenicity, as recent studies with organoids demonstrate that 5-FU is mutagenic [65]. Increased mutagenicity has implications for use in cancer treatment [66], including immunotherapy applications, which are affected by overall tumor mutational burden [67]. The mechanism by which 5-FU is mutagenic is not known; however, the characteristic mutational signature of 5-FU involves T > G substitutions in a “CTT” context, which is unexpected based on 5-FU forming base pairs primarily with A and also with G [68]. The mutational profile is consistent with 5-FU inducing mutations by causing oxidative stress with 8-oxo-dG forming base pairs with A [69] ultimately resulting in a G > T transversion. Furthermore, FdU substitution in DNA perturbs both base-excision repair (BER) [9,70] and mismatch repair (MMR) processes [70]. Specifically, FdU alters the dynamics of the base pair opening in duplex DNA, which could affect how uracil excision repair enzymes recognize and excise 5-FU from DNA [9,71,72]. The MMR complex MutSα also differentially binds base pair mismatches that include FdU, which can affect the balance between repair signaling and induction of apoptosis [73].

## 4. Inhibition of RNA Modification Enzymes and RNA-Mediated Effects of FPs

5-FU causes serious systemic toxicities in some patients including both gastrointestinal (GI) and hematopoietic effects. Clinically, high-grade 5-FU toxicities are treated with Urd triacetate [74], which is consistent with an RNA-directed mechanism for 5-FU’s most serious systemic toxicities. Specifically, 5-FU was shown to induce p53-dependent apoptosis in intestinal cells [75]. The mechanism by which 5-FU perturbs RNA-mediated processes remains under active investigation. In essentially all cells, 5-FU is readily converted to the corresponding ribonucleotide triphosphate (FUTP) and incorporated into diverse species of RNA, including tRNA, which undergoes multiple post-transcriptional modifications that may be affected by 5-FU substitution. In particular, ribothymidine (rT) and pseudouridine (Ψ) are Urd modifications that are found in the “TΨC” loop common to all tRNAs. Furthermore, Ψ is a highly conserved modification at other sites in tRNA and in multiple RNA species. Substitution of 5-nitrouridine in tRNA was shown to trap tRNA-Ura methyl transferase (RUMT; [76]) by the formation of a reversible covalent complex in which Cys324 of RUMT forms a Michael adduct with NO_2_Ura54 in tRNA [77]. The role of TRMT2A, the mammalian tRNA methyltransferase 2 homolog A protein, for mammalian cell function remains unclear; however, a recent study demonstrated that TRMT2A-deficient cells displayed increased growth consistent with a role for TRMT2A in cell cycle regulation [78]. Furthermore, other studies implicated TRM2A in DNA DSB repair in yeast [79]. These findings indicate that 5-FU could affect cell cycle and DNA repair processes in mammalian cells through the inhibition of TRMT2A [80].

Pseudouridine is sometimes referred to as a “5th nucleotide” because it is such a common RNA modification [81]. In addition to the conserved modification of tRNA in the TΨC loop of all tRNA and at conserved sites in specific tRNA, Ψ is also present in mRNA, rRNA, and snRNA. The biological function of pseudouridylation remains under investigation, but it is known that Ψ base pairs with all four major bases have greater stability than Urd [82]. Ψ formation results from isomerization of uridine with cleavage of the N-glycosidic bond and formation of a C-glycosidic bond to C5. The mechanism of pseuoduridine synthases (Pus) is distinct from tRNA methyl transferases and involves nucleophilic attack by an aspartate rather than a cysteine, possibly at C1′ rather than C6 [81,83]. There appears to be variability in the mechanism among different pseudouridine synthases, and 5-FU substitution in substrate RNA potently inhibits some, but not all of these enzymes [14].

## 5. Synthesis of 5-FU Substituted RNA and DNA for Biophysical Studies

**Figure 4:**
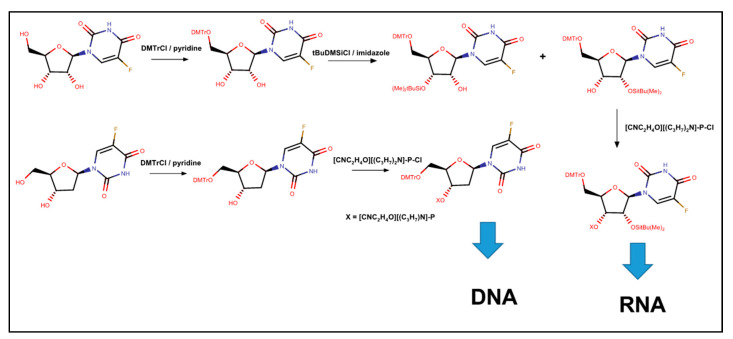
Scheme summarizing the synthesis of protected FUrd and FdU phosphoramidites for incorporation into RNA and DNA oligonucleotides. The initial step for each is the protection of 5′-OH with dimethoxytrityl chloride in dry pyridine. For RNA, protection of the 2′-OH is performed using tert-butyldimethylsilyl chloride in dry pyridine with imidazole which occurs without regiospecificity requiring purification of the 2′OH protected species. The 3′-OH was then reacted with cyanoethyl *N*,*N*-diisopropylphosphonamidic chloride in dry THF using diisopropylethylamine and a catalytic amount of dimethylaminopyridine. The resulting phosphoramidites were used in automated DNA and RNA synthesis using standard procedures.

The biological activities of 5-FU that are responsible for both its anti-tumor activity and systemic toxicities remain only partly characterized. To study the effects of FUrd and FdU substations on RNA and DNA in a sequence-specific manner required synthesis of the corresponding nucleoside phosphoramidites and their incorporation into oligonucleotides using automated DNA or RNA synthesis. The Gmeiner lab reported the synthesis of FUrd phosphoramidite [84] by adapting the protection strategy used for Urd in automated RNA synthesis (Figure 4). Briefly, this involved protecting 5′-OH with 4,4′-dimethoxytrityl chloride and 2′OH with *tert*-butyldimethylsilyl chloride. The 3′-OH was then converted to the reactive phosphoramidite using cyanoethyl *N*,*N*’-diisopropylphosphonamidic chloride. The resulting FUrd derivative was then site-specifically incorporated into RNA sequences using automated synthesis with only moderate adjustments to coupling cycles relative to the native nucleoside. The FP nucleobase required no special protection chemistry. A similar approach was adopted for FdU, except without the 2′-OH protection step [21].

The effects of 5-FU substitution on DNA and RNA stability are a net result of multiple forces that are differentially affected by the strong electronegativity of 5-FU. The pK_A_ for the imino hydrogen of 5-FU is in the physiological range [85,86]. Thus, 5-FU base pairs are anticipated to be more dynamic and potentially contribute less to nucleic acid stability than native base pairs. Furthermore, in its neutral form, the keto-enol tautomer distribution of 5-FU [87] differs from Ura due to fluorine electronegativity [88,89], which may affect base pair geometry, and indirectly affects base stacking, which is a predominate force in duplex stability [90]. The net effects of FUrd substitution in RNA and FdU substitution in DNA were found to depend on both the site of substitution and how many sites were substituted. In DNA, single FdU substitution moderately destabilized the duplex or had no effect. In RNA, single FUrd substitution moderately stabilized the duplex [91]. Two FdU substitutions in DNA moderately destabilized the duplex, but in RNA had no significant effect. NMR studies showed FdU substitution affected the base roll angle causing FdU-substituted duplexes to be bent compared to non-substituted duplexes [92]. In an RNA duplex derived from U4 snRNA, a G-FUrd base pair adopted Wobble geometry, reduced stacking for G-FUrd, and contributed to a slight decrease in duplex stability [68]. Overall, the effects of 5-FU substitution on RNA structure and stability were found to be moderate. Thus, 5-FU is also used as a probe to monitor RNA structure and dynamics using ^19^F NMR [93,94]. An enzymatic synthesis of FUTP was developed by Williamson and co-workers to facilitate RNA labeling for NMR studies [95].

The inclusion of fluorinated pyrimidines into DNA affects not only base pairing and stacking, but also effects ion binding and occupancy of the major and minor grooves of the DNA duplex. The Gmeiner lab demonstrated that Zn^2+^ binds duplex DNA containing consecutive FdU-dA base pairs in the major groove. Zn^2+^ is bound in a distorted trigonal bipyramidal geometry with O4 and F5 on consecutive FdU as axial ligands and three water molecules as equatorial ligands [96,97]. Zn^2+^ complexation inhibited ethidium bromide intercalation and stabilized the duplex by ~15 °C. In contrast, Mg^2+^ did not inhibit EtBr intercalation and displayed a lesser effect on duplex stability. While affecting electrostatics in the major groove of duplex DNA, FdU substitution minimally perturbed minor groove structure. Netropsin binds tightly to the minor groove of FdU-substituted DNA at A-T sites and binding was not disrupted by Zn^2+^ in the major groove. Interestingly, while fluorine substitution indirectly affects base pair formation in FdU-substituted DNA and alters the electrostatics in the major groove, the minimal propensity of fluorine to engage in hydrogen bond formation permitted 2,4-dinitrotoluene to serve as an isosteric thymidine analog to study the importance of hydrogen bond formation in polymerase specificity [98].

## 6. Conclusions

FPs continue to be the most widely used drugs for CRC treatment and are highly relevant for cancer treatment in the era of personalized medicine [99]. The chemistry and biochemistry of FPs continues to evolve, and their inclusion in DNA and RNA enables novel chemistry though the perturbation of base pairing, base stacking, and the ionic environment [96,97]. Our understanding of the enzymatic processes perturbed by FPs that are responsible for the biological effects of FPs, including both anti-tumor activity and systemic toxicities, also continues to evolve. The importance of TS inhibition for the anti-tumor activity of FPs was established decades ago [6,11] and has stood the test of time in clinical practice [10]. Other enzymatic processes are perturbed by 5-FU, including ribomethyltransferase [76] and pseudouridylate synthase [83], and these may affect RNA function. However, the relevance of targeting these enzymes for cancer treatment remains unproven, although new insights into alternative functions for these enzymes [78] may enable their rational targeting. More recently, Top1 was shown to be perturbed by FdU substitution [13]. Furthermore, FP-induced Top1cc displays an altered dependence on Tdp1- and PARP1-mediated repair [60] and are accentuated by TS inhibition indicating distinct properties from established Top1 poisons. Thus, there is increasing potential to design FPs to activate specific processes that contribute to anti-tumor activity while minimizing processes that contribute to systemic toxicities. FPs display strong potential for continuing prominent use in the era of personalized medicine and could provide a benefit in immunotherapy applications [66].
